# Efficacy and safety of *Maekmoondong-tang* for chronic dry cough: a study protocol for a randomized controlled trial

**DOI:** 10.1186/s12906-016-1028-x

**Published:** 2016-02-02

**Authors:** Kwan-Il Kim, Seungwon Shin, Kyuseok Kim, Junhee Lee

**Affiliations:** 1 Department of Clinical Korean Medicine, College of Korean Medicine, Kyung Hee University, 23 Kyungheedae-ro, Dongdaemun-gu, Seoul, 130-872 Republic of Korea; 2 Department of Sasang Consitutional Medicine, College of Korean Medicine, Kyung Hee University, 23 Kyungheedae-ro, Dongdaemun-gu, Seoul, 130-872 Republic of Korea; 3 Division of Allergy, Immune and Respiratory System, Department of Internal Medicine, College of Korean Medicine, Kyung Hee University, 23 Kyungheedae-ro, Dongdaemun-gu, Seoul, 130-872 Republic of Korea; 4 Department of Ophthalmology & Otolaryngology & Dermatology, College of Korean Medicine, Kyung Hee University, Seoul, Republic of Korea

**Keywords:** Chronic dry cough, *Maekmoondong-tang*, Efficacy, Safety, Herbal medicine

## Abstract

**Background:**

Chronic cough, defined it lasts more than 8 weeks. The symptom is common, but highly troublesome, and it reduces quality of life. Despite much effort to develop a protocol for diagnosis and treatment of chronic cough, it remains problematic to determine its cause. As a result, treatment is often unsuccessful. Thus, there is much interest regarding the use of symptomatic drugs to control chronic cough.

*Maekmoondong-tang* is widely used in East Asian countries to treat chronic dry cough. Several experimental studies have reported that the herbal medicine has immunomodulatory and antitussive effects. Clinical studies involving *Maekmoondong-tang* have also been carried out; however, these studies have involved treating various diseases as a whole rather than chronic cough itself. Thus, we aim to evaluate the efficacy and safety of *Maekmoondong-tang* in chronic dry cough patients with a randomized controlled trial.

**Methods/Design:**

This study is designed as an exploratory, single-center, placebo-controlled, double-blind, randomized, parallel group clinical trial. Patients with dry cough that has lasted more than 8 weeks will be recruited, after a 1-week run-in period, and randomly allocated to either the *Maekmoondong-tang* treatment group or the placebo group. The patients will receive *Maekmoondong-tang* or placebo granules 3 times daily for 4 weeks, with a 2-week follow-up. The primary outcome is a 10-point cough diary that will be recorded on a daily basis. The secondary outcomes comprise a cough visual analog scale, the Leicester Cough Questionnaire (Korean version), the Pattern Identification for Chronic Cough Questionnaire, biomarkers, safety testing, etc. Adverse events will also be reported.

**Discussion:**

This trial will assess the efficacy and safety of *Maekmoondong-tang* in chronic dry cough.

**Trial registration:**

Korean Clinical Trial Registry (http://cris.nih.go.kr; registration number: KCT0001646). Date of registration: October 5 2015

## Background

Chronic cough, defined as lasting more than 8 weeks, is a common respiratory symptom among outpatients [1–3]. It is associated with a substantial deterioration in quality of life, with various effects on all aspects of health, such as retching, vomiting, chest pain, rib fracture, incontinence, fainting, and depression [2, 4]. The management and treatment of chronic cough is often unsatisfactory, although a few clinical guidelines are available [5]. The most common conditions that lead to chronic cough are asthma, upper airway cough syndrome, and gastroesophageal reflux disease with a normal chest radiograph [3]. However, in up to 46 % of patients, no single cause is evident [6], and the treatment success rate has been reported to be as low as 58 % [7]. The number of patients with unexplained cough who do not improve after cause-specific treatment is increasing; therefore, clinicians and patients alike are concerned about cough treatment [5]. Despite this, studies into the efficacy of antitussive drugs to manage chronic cough have yielded inconsistent results. Moreover, the agents used showed adverse effects [8], and evidence for the efficacy of inhaled corticosteroids (ICS), which are recommended for chronic cough, is not strong [9]. Therefore, a limited range of drugs is available to care chronic cough.

*Maekmoondong-tang* (MMDT; *Mai-men-dong-tang* in Chinese; *Bakumondo-to* or TJ-29 in Japanese) was introduced in the Geumgweyoryak, a classical textbook of traditional Chinese medicine [10]. MMDT, which consists of six herbs, is widely used to treat cough caused by lung *yin* deficiency, which is a common pattern in chronic dry cough according to traditional Korean medicine (TKM) [11]. Here, “*yin*” refers in general to body fluids, and “lung *yin* deficiency” denotes a shortage of *yin* of the lung; that is, a dry lung. This lung *yin* deficiency is manifested by unproductive cough, afternoon fever, night sweating, flushed cheeks, dry throat, red and dry tongue, etc. [12]. According to the Donguibogam, which was published in 1613 by the royal physician Heo Jun and has been a popular piece of TKM literature, MMDT is also used for heat-induced asthma, which is caused by the lung or stomach *yin* deficiency [13]. The immunomodulatory [14] and anti-asthmatic [15, 16] effects of MMDT have been investigated in several studies. A peripheral antitussive effect of MMDT has also been reported [17, 18]; that is, the drug can be used to treat coughs that are not controlled by central cough suppressants like codeine. The results of case–control clinical studies have shown that MMDT reduces cough hypersensitivity [19, 20], which is another recently proposed cause of chronic cough [21, 22]. MMDT clinical trials have been carried out, but have focused only on specific diseases, such as asthma [23], chronic obstructive pulmonary disease (COPD) [24], and post-infectious cough [25, 26], not on the symptom itself. To our knowledge, no clinical trials have studied the efficacy of MMDT on chronic dry cough with normal chest X-ray.

Therefore, we aim to investigate the efficacy and safety of MMDT for chronic dry cough. We will evaluate cough symptoms in terms of their intensity and severity using a cough diary, cough visual analog scale (VAS), and cough-specific quality of life questionnaire.

## Methods/Design

### Objective

The objective of this trial is to assess the efficacy and safety of MMDT for the adult patients with chronic dry cough.

### Hypothesis

We primarily hypothesize that 4 weeks of MMDT intervention eases chronic dry cough in adults more efficaciously than a placebo control. We will evaluate this hypothesis using a 10-point cough diary.

### Design

**Fig. 1:**
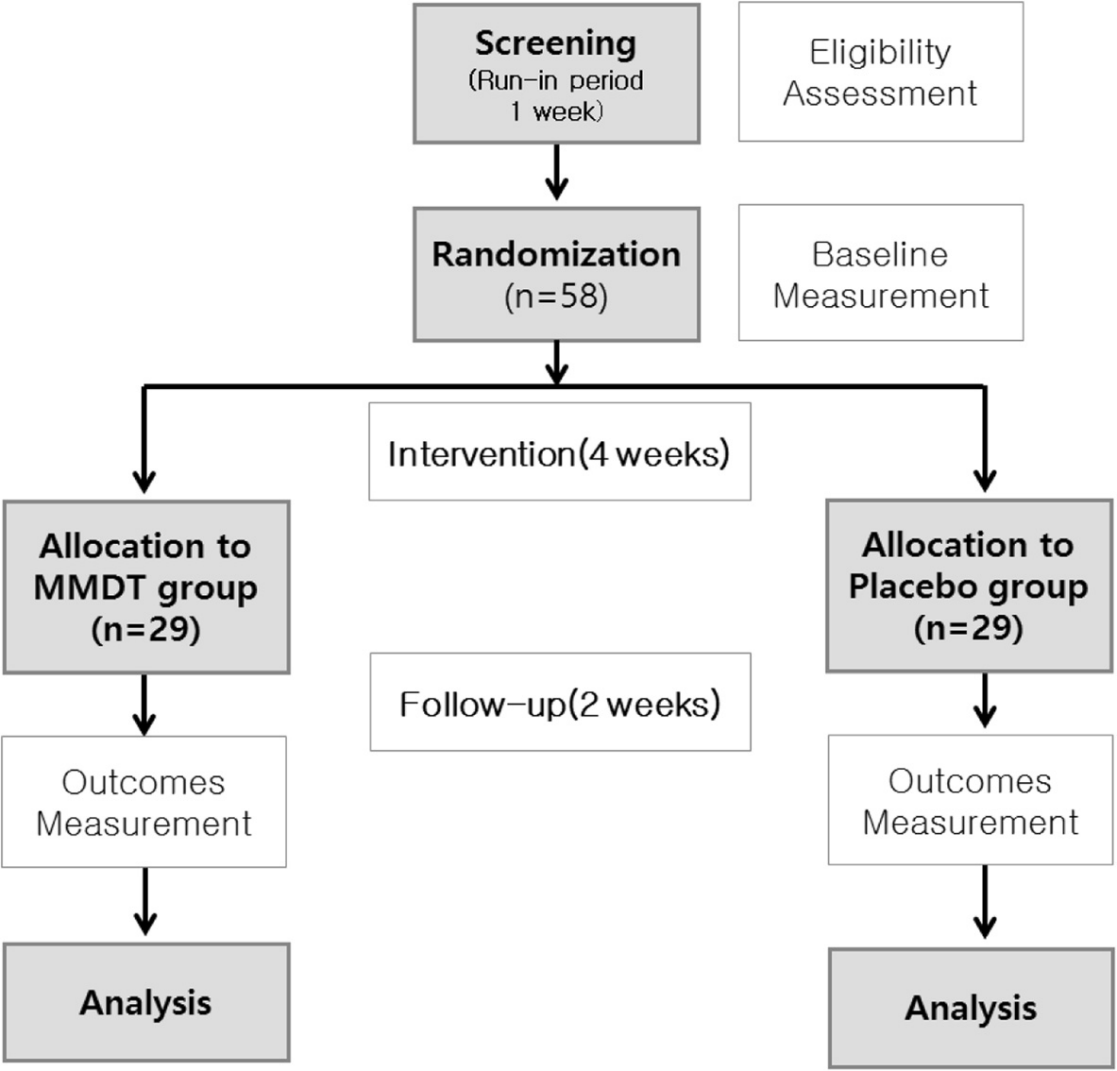
Study flow chart

This study is an exploratory, single-center, placebo-controlled, double-blind, randomized, parallel group clinical trial. Figure 1 shows a flow chart of the study. Eligibility for the study will be decided on the basis of a daily cough diary, which participants will keep during the 1-week run-in period. Those with a cough diary symptom score of more than 2, as well as more than 10 entries in the cough diary during the run-in period, will be enrolled pending the inclusion and exclusion criteria below.

### Participants

#### Inclusion, exclusion, and withdrawal criteria

The inclusion criteria are: (1) age between 19 and 75 years; (2) chronic cough that has lasted more than 8 weeks; (3) dry cough (sputum frequency of 5 or fewer times per day, and sputum volume less than 10 ml per day, on the basis of the Sputum Severity Evaluation Standard of the Korean Ministry of Food and Drug Safety); (4) provision of written informed consent.

The exclusion criteria are: (1) abnormal pulmonary function test (forced vital capacity, or forced expiratory volume in 1 s, < 80 % of predicted); (2) abnormal chest X-ray; (3) acute respiratory disease, including upper respiratory tract infection, during the previous 4 weeks; (4) chronic pulmonary diseases (COPD, idiopathic pulmonary fibrosis, bronchiectasis, etc.) during the previous 2 years; (5) diagnosis of malignant tumor during the previous 5 years; (6) history of smoking (≥20 packs [400 cigarettes] during the participants’ lifetime); (7) treatment taking an angiotensin-converting-enzyme inhibitor at present or during the previous 4 months; (8) current or the precious 2-week use of antitussive drugs, glucocorticoids, leukotriene receptor antagonists, anticholinergic drugs, long-acting β2 agonists or any herbal medication; (9) antihistamine treatment during the previous 3 days; (10) aspartate aminotransferase (AST) or alanine aminotransferase (ALT) levels at least twofold higher than the upper normal limit, or a serum creatinine levels at least 1.2-fold the upper normal limit; (11) a mean cough diary score of less than 2 during the 1-week run-in period; (12) fewer than 10 entries in the cough diary during the 1-week run-in period; (13) pregnancy or breastfeeding; (14) illiteracy or cognitive impairment; (15) unsuitability as judged by the investigators.

The rejection and withdrawal criteria are: (1) treatment that might influence the results of the trial without the agreement of an investigator; (2) use of forbidden drugs, such as antitussives, glucocorticoids, leukotriene receptor antagonists, anticholinergics, and long-acting β2 agonists; (3) participants not following the protocol, or taking < 80 % of the prescribed doses; (4) a serious adverse event (SAE) during the trial; (5) voluntary withdrawal from the trial; (6) unsuitability as judged by the investigators.

### Recruitment

Through advertisements and referrals, a total of 58 patients with chronic dry cough will be recruited at the Korean Medicine Clinical Trial Center (K-CTC) of the Kyung Hee University Korean Medicine Hospital.

### Ethics

This trial has been authorized by the Institutional Review Board of the Kyung Hee University Korean Medicine Hospital (approval number: KOMCIRB-150213-HRBR-007). The protocol accords with both the Declaration of Helsinki and Good Clinical Practice (GCP) Guidelines. Signed informed consent forms will be obtained from all eligible participants before enrollment.

This trial is registered with the Korean Clinical Trial Registry (registration number: KCT0001646).

### Randomization and Concealment

An independent statistician who is unware of the design and purpose of the study will generate a randomization table using R software (version 3.2.0; The R Foundation for Statistical Computing); specifically, a block size of 4 will be used, and the 58 patients will be randomly allocated group in a 1:1 ratio either to the MMDT treatment group or to the placebo control. The statistician will keep the randomization lists and inform the researcher of the matching cord number either by text message or via mobile communication application.

### Blinding

Participants, investigators, and the clinical trial pharmacist will be blinded to the treatment allocation throughout the course of the study. The placebo granules will be similar to the MMDT granules in appearance, taste, and scent. The manufacturer will label the random and product codes on the packaging, and the code labeling will conform to the GCP guidelines. The clinical trial pharmacist at the K-CTC will provide the packaged drugs to the patients on the basis of the randomization number. The statistician will uncover the blinding when necessary, such as an SAE occurs. A blinding test will be conducted to evaluate the success of blinding after 4 weeks of intervention.

### Intervention

#### MMDT

The MMDT group will receive MMDT granules (3 g/pouch, 3 times per day—before each meal—for 4 weeks; dosage based on the requirements of the Korean Ministry of Food and Drug Safety). The MMDT granules are manufactured as “Maekgeuron Granules” by Hanpoong Pharm & Food Co. Ltd. (Jeonju, Republic of Korea), a company that has obtained Korea Good Manufacturing Practice authorization. Both the MMDT granules and their ingredients have been approved by the Korean Ministry of Food and Drug Safety. Three grams (dry weight) of granules (water-extracted MMDT combined with starch and lactose) contain six herbs: *Ophiopogonis* Tuber (*Liriope platyphylla* Wang et Tang, family Liliaceae; 3.33 g), *Pinelliae* Tuber (*Pinellia ternata* Breitenbach, family Araceae; 1.67 g), *Glycyrrhizae* Radix (*Glycyrrhiza uralensis* Fischer, family Leguminosae; 0.67 g), *Zizyphi* Fructus (*Zizyphus jujuba* Miller var. *inermis* Rehder, family Rhamnaceae; 1.00 g), *Ginseng* Radix (*Panax ginseng* C. A. Meyer, family Araliaceae; 0.67 g), and *Oryzae* Semen (*Oryza sativa* Linné, family Gramineae; 3.33 g). This 10 g mixture yields 2.07 g of soft extract after boiling in water. With starch and lactose added, the final MMDT of 3 g is obtained by drying under reduced pressure. Each 3 g dose of MMDT contains 3.1 mg of glycyrrhzin acid and 0.31 mg of gincenoside Rb1 (Rg1). Voucher specimens will be reserved at the research library of Hanpoong Pharm & Food Company.

#### Placebo

The control group will receive placebo granules (3 g/pouch, 3 times per day—before each meal—for 4 weeks). The placebo was manufactured by the Jeonnam Bioindustry Foundation Food Research Center (JBF), following the placebo guidelines of the Korean Ministry of Food and Drug Safety. The granules do not contain any active ingredients, but are similar in appearance, taste, and scent to the MMDT granules.

All products were packaged by JBF (Naju, Republic of Korea). Either Forty-two MMDT or placebo pouches will be provided to each randomized participant at visit 1 (week 0 ± 3 days) and visit 2 (week 2 ± 3 days). The products will be stored at the K-CTC clinical research pharmacy in Kyunghee University Korean Medicine Hospital, and an independent and trained pharmacist will manage all procedures associated with the drugs. The study process is outlined in Table 1.

**Table 1:** Study process: treatment and outcome measurements

Activity	Screening	Visit 1	Visit 2	Visit 3	Follow up
Time schedule	-Week 1	Baseline	Week 2	Week 4	Week 6
Window visit		±3 days	±3 days	±3 days	±3 days
Informed consent	X				
Demographics	X				
Cough symptom check	X				
Medical history	X				
Smoking history	X				
Medication history	X				
Safety test	X	X		X	
Chest X-ray	X				
Pulmonary function test	X				
Safety test	X				
Methacholine bronchial challenge test^a^	X				
Nasal endoscopy^a^	X				
Distribute cough diary card	X	X	X	X	
Randomization		X			
Height/weight/body mass index		X			
Vital sign		X			
Cough diary score		X	X	X	X
Cough visual analogue score		X	X	X	X
Leicester cough ques-tionnaire Korean versi- on		X		X	X
Pattern identification for chronic cough question-naire		X		X	
Cold-Heat pattern questionnaire		X		X	
Yin deficiency scale		X		X	
Hematological biomarker		X		X	
Immunologic biomarkers		X		X	
Investigational Produce		X	X		
Compliance			X	X	
Adverse events investi-gation			X	X	
Concomitant therapy eva- luation	X	X	X	X	

^a^The test will be performed as needed, according to the judgment of the investigator

### Concomitant and forbidden drugs

If participants experience unbearable cough at night, taking one capsule acetylcysteine is permitted; this antitussive will be delivered to the patients along with the MMDT or placebo granules. If this does not improve the cough, the participant will visit the K-CTC for examination and appropriate management. The participants will be asked to record whether they take the antitussive agent or not. Other drugs that alleviate cough—such as other antitussives, glucocorticoids, leukotriene receptor antagonists, anticholinergics, short-acting β2 agonists, long-acting β2 agonists, and antihistamines—are prohibited. Drugs which are not related to cough symptoms will be permitted. The name, duration, and dosage of any other drugs taken will be recorded in the case report forms.

### Outcome measures

#### Primary outcomes

The primary outcome is the cough diary score at week 4. The cough diary score on the last day of the 1-week run-in period will be taken as the baseline score of participants who will be enrolled and randomized for the trial.

The cough symptom scoring recommended by Spector et al. will be used in this study [27]. The daily cough diary consists of two parts: daytime (08:00–20:00) and nighttime (20:00–08:00) cough symptoms. Patients will be required to evaluate their symptoms twice per day (daytime and nighttime). Daytime cough symptoms will be graded from zero to five as follows: 0—no cough; 1—cough for one short period; 2—cough for two or more short periods; 3—frequent coughs that did not interfere with usual daytime activities; 4—frequent coughs that did interfere with usual daytime activities; and 5—distressing coughs for most of the day. Nighttime cough symptoms will be graded from zero to five as follows: 0—no cough during the night; 1—cough on waking only; 2—waking once or early precisely because of cough; 3—waking frequently due to cough; 4—wakefulness for most of the night due to cough; and 5—distressing coughs preventing sleep. The total cough score (from 0 to 10) is the sum of the daytime and nighttime cough symptom scores.

#### Secondary outcomes

##### Cough visual analog scale

The cough VAS is an overall rating scale for cough frequency and severity ranging from 0 (no cough) to 10 (unbearable cough). The scale will be checked at baseline, and at weeks 2, 4, and 6 (follow-up visit). The cough VAS mean scores will be recorded at weeks 2, 4, and 6.

##### The Leicester Cough Questionnaire (Korean version)

The Leicester Cough Questionnaire (LCQ) is widely used to measure quality of life in cough patients. It was developed by Birring [28], and has been validated in several languages, including Korean (LCQ-K) [29]. The LCQ comprises three domains: physical, mental, and social quality of life; a total of 19 items are scored on 7-point Likert scales. A higher score indicates a more healthy state. Several studies [2, 30, 31] have demonstrated a correlation between cough symptom severity and LCQ score. At week 4, we will compare the MMDT and placebo groups in terms of mean LCQ-K scores.

##### Pattern Identification for Chronic Cough Questionnaire

The Pattern Identification for Chronic Cough Questionnaire (PICCQ) is used to identify patterns in chronic cough patients. We developed this questionnaire using the Delphi method [32], and conducted clinical research to confirm its validity and reliability. The PICCQ consists of four patterns: wind-cold, phlegm-turbidity, fire-heat, and deficiency (lung deficiency and kidney yang deficiency). The PICCQ comprises 38 items scored on 5-point Likert scales. A patient’s pattern is calculated by summing the scores of each item. We will investigate the chronic cough pattern distribution in chronic dry cough patients, as well as the correlation between chronic cough pattern and the efficacy of MMDT.

##### Cold-Heat Pattern Questionnaire

We will use the validated Cold-Heat Pattern Questionnaire, which consists of 20 symptom items (10 items to assess cold pattern and 10 to assess heat pattern) [33]. Each item requires only a “yes” or “no” response depending on the patient’s tendencies during the previous week. The total numbers of items that a participant responds ‘yes’ will be the cold or heat pattern score, respectively, for the responder. We will investigate the Cold-Heat Pattern in chronic dry cough patients, as well as the correlation between the cold-heat pattern scores and the efficacy of MMDT.

##### Yin Deficiency Scale

We will use the validated *yin* deficiency scale developed by Park [34]. This questionnaire consists of 27 items, each of which uses a 7-point Likert scale; the cut-off point was defined as 10 points. We will use the *yin* deficiency scale to evaluate chronic dry cough patients, as well as to investigate the correlation between the *yin* deficiency pattern and the efficacy of MMDT.

### Biomarkers

Biomarkers that reflect chronic cough and airway inflammation [35] will be evaluated. These include blood neutrophils, eosinophil counts, serum immunoglobulin E levels, sputum eosinophilic cationic protein levels, and proinflammatory cytokines such as tumor necrosis factor alpha, interleukin (IL)-4, IL-5, IL-8, IL-10, and IL-13.

### Adverse event reporting

An adverse event (AE) is an undesirable, unintended sign, symptom, or disease that does not necessarily have a cause-and-effect relationship with the intervention evaluated in a clinical trial. We will carry out continuous monitoring of AEs and make any decision in this regard on the basis of both objective and subjective signs, as well as blood test results. Appropriate measures will be taken immediately to minimize any SAEs.

### Safety outcomes

Safety will be investigated using adverse reaction reports and clinical laboratory tests; namely, liver function tests (AST, ALT, alkaline phosphatase [ALP], total bilirubin [TB], and γ-glutamyltranspeptidase [GGT] levels), renal function tests (blood urea nitrogen [BUN] and creatinine levels), and serum sodium and potassium levels. All women of childbearing age will also undergo human chorionic gonadotropin testing.

### Sample size calculation

We referred a randomized controlled trial (RCT) that was conducted to demonstrate the clinical efficacy of a herbal medication for the patients with cough variant asthma (CVA) [36]. It was inevitable because no RCTs have been carried out that address the comprehensive chronic cough symptom using a herbal mediation. Furthermore, the main manifestation of CVA is cough lasting more than 8 weeks without any abnormal pulmonary functions; this is very similar to clinical characteristics of the eligible participants recruited in this study.

Using a cough diary score, the CVA study compared the efficacy of a herbal medicine with that of standard therapy (leukotriene receptor antagonist combined with methylxanthine) for 2 weeks. The calculated effect size was 0.699 (α = 0.05, 1-β = 0.8, 2-tailed test). Because of the discrepancy in comparators and study duration between the previous and planned studies, we determined to eliminate the effect of standard therapy and add the effect of longer treatment duration; this resulted in an adjusted effect size of 0.85. Assuming a 20 % dropout rate, and an allocation ratio of 1, we have calculated the necessary sample size to be 58 participants (29 in each group). The above calculations were performed using G*Power™ software (ver. 3.1.9.2) for Mac.

### Statistical analysis plan

Data entry and management will be completed by an independent data administrator to ensure data accuracy. We will analyze the efficacy and safety of MMDT using the “intent-to-treat” (ITT) principle. Subordinately, we will also present the results of the analysis based on the “per protocol” principle.

#### Patient groups for data analysis

##### Intent-to-treat population

The ITT population will include all participants treated with at least one dose of the study drug and who keep and return a daily cough diary for at least one day of recordings.

##### Per-protocol population

The per-protocol population will include patients who take more than 80 % of the prescribed doses of the study drug, fill in the daily cough diary on 80 % of the study days, and return their diary to the researchers.

##### MMDT responder/non-responder groups

An MMDT treatment responder is defined as a participant who exhibits a 75 % decrease in cough diary score after 4 weeks of treatment; all other participants in the MMDT treatment group will be included in the non-responder group. We will analyze the correlations between MMDT responder group and all TKM patterns.

### Statistical analysis

Descriptive statistics will be used for continuous variables, and frequencies for categorical variables. The “last observation carried forward” (LOCF) principle will be used to compensate for missing data. The LOCF principle involves replacement of missing data with the last observed value to obtain a complete database.

When the assumption of normality is satisfied will a *t*-test be used to compare the two groups in terms of the primary outcome (mean cough diary score at week 4). Otherwise, the Mann–Whitney *U*-test will be used. Other continuous variables, such as mean efficacy measurements (cough VAS score or LCQ scores), safety tests (AST, ALT, ALP, TB, GGT, BUN, creatinine, sodium or potassium levels), and biomarkers will be analyzed using the same statistical methods. Associations between the MMDT responders and non-responders with regard to each identified pattern will be estimated using a logistic regression model, which will be adjusted for covariates. All data will be analyzed using SPSS™ for Windows software. The level of significance will be set at a two-sided P value of 0.05.

We, along with a professional statistician, will perform the data analysis for the results.

## Discussion

Chronic cough is a common symptom in respiratory outpatients, and in an increasing number, the symptom is not eased by conventional treatments. Recent studies have found that substantially fewer patients now respond fully to treatment [7, 37, 38]. Due to the increased patients and limited treatment options, chronic cough is considered a challenging clinical problem.

MMDT is a traditional herbal medicine that has long been used to treat chronic dry cough; however, the evidence regarding the medicine’s efficacy is sparse. We aim to demonstrate the efficacy and safety of MMDT in chronic dry cough via a randomized, placebo-controlled clinical trial that has been designed in accordance with the Consolidated Standards of Reporting Trials guidelines [39]. In addition, we will investigate the relationship between the specifically identified pattern of cough and the effectiveness of MMDT. This trial will provide high-quality evidence on the efficacy and safety of MMDT in the treatment of chronic dry cough. A larger-scale clinical trial to evaluate the effectiveness and safety of MMDT in treating chronic dry cough, based on this study and with specific cough patterns identified, will be possible in future.

### Trial status

This clinical trial was reviewed by the Ethics Committee of Kyung Hee University Korean Medicine Hospital in March 2015. The trial started in September 2015 and is recruiting patients.

## Abbreviations

AE: adverse event
ALP: alkaline phosphatase
ALT: alanine aminotransferase
AST: aspartate aminotransferase
BUN: blood urea nitrogen
COPD: chronic obstructive pulmonary disease
CVA: cough variant asthma
GGT: γ-glutamyltranspeptidase
IL: interleukin
ITT: intent-to-treat
JBF: Jeonnam bioindustry foundation food research center
K-CTC: Korean medicine clinical trial center
GCP: good clinical practice
LCQ: Leicester cough questionnaire
LCQ-K: Leicester cough questionnaire (Korean version)
LFTs: liver function tests
LOCF: last observation carried forward
MMDT: *Maekmoondong-tang*
PICCQ: pattern identification for chronic cough questionnaire
RFTs: renal function tests
RCT: randomized controlled trial
SAE: serious adverse event
TB: total bilirubin
TKM: traditional Korean medicine
VAS: visual analog scale
